# Analysis of Biochemical and Clinical Pregnancy Loss Between Frozen-Thawed Embryo Transfer of Blastocysts and Day 3 Cleavage Embryos in Young Women: A Comprehensive Comparison

**DOI:** 10.3389/fendo.2021.785658

**Published:** 2021-12-24

**Authors:** Xiuliang Dai, Tingting Gao, Xiyang Xia, Fang Cao, Chunmei Yu, Tianfu Li, Lingjun Li, Yufeng Wang, Li Chen

**Affiliations:** Department of Reproductive Medicine Center, The Affiliated Changzhou Maternal and Child Health Care Hospital of Nanjing Medical University, Changzhou, China

**Keywords:** embryo developmental stage, biochemical pregnancy loss, clinical pregnancy loss, frozen-thawed embryo transfer, young women

## Abstract

**Background:**

To determine whether the embryo developmental stage affects biochemical or clinical pregnancy loss in young women undergoing frozen-thawed embryo transfer (FET) and to investigate the underlying mechanism.

**Methods:**

This was a retrospective study including a total of 18,34 β-HCG (human chorionic gonadotropin)-positive FET cycles. According to the morphological appearance (MA) of transferred blastocysts, FET cycles with blastocysts were divided into two groups: Group A: morphologically good (MG) blastocysts only, and Group B: at least one morphologically non-good (MNG) blastocyst. FET cycles with day 3 cleavage embryos were assigned as Group C. Biochemical and clinical pregnancy loss were the main outcome measures.

**Result(s):**

We predicted 78% *in vivo*-formed MG and 53.9% *in vivo*-formed day 5 blastocysts in Group C. (a) Including cases in Group A and Group B for binary logistic regression, we showed that Group B and day 6 blastocysts had significantly higher rates of BPL and CPL than Group A and day 5 blastocysts, respectively. (b) Including cases in Group A, Group B, and Group C for binary logistic regression, we showed that Group C had a significantly higher rate of BPL than Group A and day 5 blastocysts and a similar rate of BPL as Group B and day 6 blastocysts. Group C had a higher rate of CPL than Group A (p=0.071) and day 5 blastocysts (p=0.039), and a lower rate of CPL than Group B (p=0.199) and day 6 blastocysts (p=0.234).

**Conclusion(s):**

(1) MA and days of usable blastocysts could serve as independent factors affecting the occurrence of BPL and CPL. (2) Transfer of day 3 cleavage embryos may produce “unusable blastocysts” *in vivo*, which significantly increased the rate of BPL. (3) The rate of CPL resulting from the transfer of day 3 embryos may depend on the rate of *in vivo*-formed MG or day 5 blastocysts. Our study indicated that the difference in the BPL or CPL between transfer of blastocysts and day 3 cleavage embryos may largely depend on the quality of embryos transferred.

## Introduction

The physiological process of pregnancy begins with the event of fertilization. The fertilized zygote develops into a Day 3 cleavage embryo, which further becomes a blastocyst. Then, the blastocyst initiates the embryo–utero interaction and secretion of β-HCG (1). A positive serum β-HCG test indicates biochemical pregnancy (2). With successful implantation and subsequent further development, gestational sacs can be found by ultrasound examination, indicating the achievement of clinical pregnancy. However, biochemical pregnancy loss (BPL) or clinical pregnancy loss (CPL) often occurs during pregnancy, resulting in a failed pregnancy (3, 4). Previously, Day 3 cleavage embryos were the most commonly used for transfer in *in vitro* fertilization (IVF). In recent years, extended culture of Day 3 embryos to the blastocyst stage has been widely adopted in most reproductive centers worldwide. The transfer of blastocysts can achieve better clinical outcomes (5). One of the most important reasons for this is that extended culture can eliminate embryos with low developmental potential (DP) (6, 7). In addition, would blastocyst transfer result in reduced pregnancy loss (BPL or CPL) compared to Day 3 cleavage embryo transfer?

However, few studies have been designed to compare the rate of BPL or CPL between the transfer of Day 3 embryos and blastocysts. Two previous systematic reviews with low evidence indicated that the rates of CPL resulting from the transfer of Day 3 embryos and blastocysts were similar (8, 9). Opposite results were reported regarding the rate of BPL between transfer of Day 3 embryos and blastocysts. Zeadna et al. indicated that the developmental stage of embryos is associated with the occurrence of BPL (10). A recent study conducted by Alberto et al. showed a similar rate of BPL between transfer of Day 3 embryos and blastocysts (11). Therefore, this issue needs to be further investigated.

Commonly, according to the morphological appearance (MA), blastocysts can be classified into three types: morphologically good (MG) blastocysts with high DP, morphologically non-good (MNG) blastocysts with relatively low DP, and morphologically bad (MB) blastocysts with very limited DP (MB blastocysts will be abandoned in most reproductive centers) (12). In addition to the morphological score, blastocyst days are also an important indicator reflecting the DP of blastocysts (13, 14). According to the days of blastocysts, blastocysts can also be classified into three types: Day 5 blastocysts with high DP, Day 6 blastocysts with relatively low DP, and Day 7 blastocysts with very limited DP (in clinical practice, very few embryos will be cultured *in vitro* for 7 days).

For transfer of Day 3 embryos, a confirmation of biochemical pregnancy indicates that the transferred Day 3 embryo successfully reached the blastocyst stage. However, we do not know what kind of blastocyst it is *in vivo*. There is no doubt that transfer of Day 3 embryos may result in different qualities of blastocysts (MG/MNG or Day 5/Day 6 blastocysts) *in vivo* for implantation and subsequent development. However, for blastocyst transfer, blastocysts (MG/MNG or Day 5/Day 6) can be selected subjectively. The key point that helps to clarify whether there is a difference in the rate of BPL or CPL between transfer of Day 3 cleavage embryos and blastocysts is to investigate whether the quality of blastocysts (mainly reflected by MA or days of blastocysts) affects the rate of BPL or CPL. If the quality of blastocysts transferred has an impact on the rate of BPL or CPL, the expected rate of BPL or CPL from transfer of Day 3 embryos may be in the middle of the BPL or CPL from transfer of good and non-good quality of blastocysts; or, we expect a similar rate of BPL or CPL between transfer of blastocysts and Day 3 cleavage embryos.

In the present study, we determined whether the quality of blastocysts (MA or days of blastocysts) affects the rate of BPL or CPL. Then, we compared the rate of BPL or CPL among transfer of Day 3 embryos, transfer of “good” quality blastocysts, and transfer of non-good quality blastocysts. Our study may advance our understanding of the occurrence of BPL and CPL between transfer of blastocysts and Day 3 cleavage embryos.

## Materials and Methods

### Study Design

**Figure 1:**
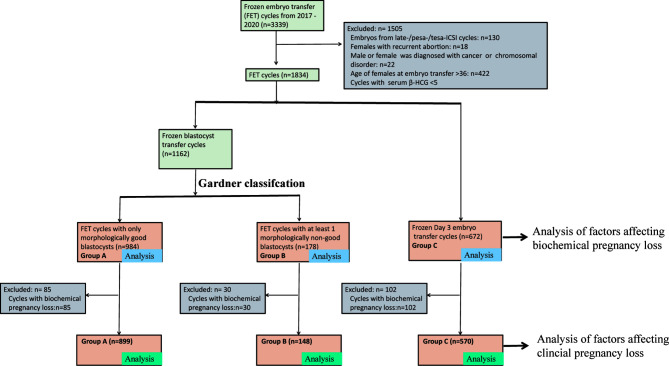
A flow chart of the study design. We initially included all FET cycles that were performed in Changzhou Maternal and Child Health Care Hospital from 2017 to 2019. According to the exclusion criteria, we excluded 1,505 FET cycles. The remaining 1,834 cycles were divided into three groups: Group A: FET cycles with only MG blastocysts; Group B: FET cycles with at least one MNG blastocyst; and Group C: Day 3 cleavage embryo transfer. Cases in Group A, Group B, and/or Group C were used to analyze factors affecting BPL. We further excluded FET cycles with BPL, and cases in Group A, Group B, and/or Group C were used to analyze factors affecting CPL. BPL, biochemical pregnancy loss; CPL, clinical pregnancy loss. FET, frozen-thawed embryo transfer.

This was a retrospective study. The data generated in the present study were collected from January 2017 to May 2020 in the reproductive center of Nanjing Medical University Affiliated Changzhou Maternal and Child Health Care Hospital. FET cycles with biochemical pregnancy were included for analysis. According to the developmental stage of embryos and morphological score of blastocysts, FET cycles were divided into three groups: FET cycles with only MG blastocysts (Group A); FET cycles with at least one MNG blastocyst (Group B); and FET cycles with Day 3 cleavage embryos (Group C). A total of 1,834 β-HCG-positive FET cycles were included. The baseline characteristics of FET cycles and patients were compared. The primary measurements were BPL and CPL. A detailed flowchart of the present study design is presented in **Figure 1**.

### Inclusion/Exclusion Criteria

- FET cycles with biochemical pregnancy (serum β-HCG >5) were included.
- Cycles with embryos from late-/pesa-/tesa-ICSI cycles were excluded.
- Females with recurrent abortion or couples in which either the male or female was diagnosed with cancer or chromosomal disorder were excluded from the analysis.
- Age of females at embryo transfer <=36. Commonly, females at 35 years old may experience a sharp reduction in the quantity and quality of oocytes. Age may be the most important factor affecting pregnancy loss for women with advanced age. Furthermore, women with advanced age undergoing IVF/ICSI treatment may have a relatively small number of available embryos for extended culture. Therefore, in the present study, we focused on young patients.

### Embryo Culture Procedures

Controlled ovarian stimulation protocols, including the long protocol, antagonist protocol, PPOS protocol, and mini-stimulation protocols, were used as previously described (15). Thirty-six hours after the trigger, oocytes were retrieved. Oocytes were fertilized by conventional IVF or the ICSI method. G1 and G2 plus medium (Vitrolife, Sweden) was used for embryo culture. Fertilization was evaluated on Day 1, and embryos were morphologically graded on Day 3 and Day 5/6.

### Morphological Score of Embryos

Day 3 cleavage embryos were scored as in a previous study (16). Blastocysts were evaluated based on the degree of blastocyst expansion, inner cell mass (ICM), and trophectoderm (TE) (12). The details of scoring for Day 3 cleavage embryos are described in **Table 1**, and those for blastocysts are described in **Table 2**. In our reproductive center, for Day 3 cleavage embryos, grade I and grade II embryos were taken as good cleavage embryos, grade III embryos were taken as non-good embryos, and grade IV embryos were abandoned.

**Table 1:** Morphological score of Day 3 cleavage embryos.

	Number of blastomeres	Size of blastomeres	Fragmentation
Grade I	8–10	Even	<10%
Grade II			
a	6–7 or >10	Even	0
b	8–10	Even	10–20%
Grade III	>4	Uneven	20–50%
Grade IV	>4	Uneven	>50%

**Table 2:** Morphological score of blastocyst.

	Score	Description
**Degree of expansion and hatching status**	1	A blastocyst with a blastocoel that is less than half of the volume of the embryo
	2	A blastocyst with a blastocoel that is half of or greater than half of the volume of the embryo
	3	A full blastocyst with a blastocoel completely filling the embryo
	4	An expanded blastocyst with a blastocoel volume larger than that of the early embryo, with a thinning zona.
	5	A hatching blastocyst with the trophectoderm starting to herniate though the zona
	6	A hatched blastocyst, in which the blastocyst has completely escaped from the zona.
**ICM**	A	Tightly packed, many cells
	B	Loosely grouped, several cells
	C	Very few cells.
**TE**	A	Many cells forming a cohesive epithelium
	B	Few cells forming a loose epithelium
	C	Very few large cells

For blastocysts, ***MG*** blastocysts were defined as blastocysts with a degree of blastocyst expansion over 3 and an A or B score of ICM and TE.

***MNG*** blastocysts were defined as blastocysts with a degree of blastocyst expansion over 3, a C score of ICM or TE, and a corresponding A or B score of TE or ICM.

***MB*** blastocysts were defined as blastocysts with ICM and TE C scores.

***Day 7 *** blastocysts were defined as the formation of MG or MNG blastocysts on Day 7.

***Usable*** blastocysts were defined as MG or MNG blastocysts on Day 5 or on Day 6.

***Unusable*** blastocysts were defined as MB blastocysts or Day 7 blastocysts.

In our reproductive center, (a) no embryo is cultured for 7 days; (b) blastocysts with a degree of blastocyst expansion <=3 are abandoned; and (c) only usable blastocysts are transferred. In addition, morphological evaluation of embryos in our reproductive center was performed by YW, who has more than 10 years of work experience as an embryologist.

### Biochemical and Clinical Pregnancy

Serum β-HCG was measured 11 or 14 days after frozen Day 3 cleavage embryo or blastocyst transfer, respectively. Biochemical pregnancy was defined as serum β-HCG >5. Clinical pregnancy was confirmed by the presence of a gestational sac with fetal heartbeat (14 days after biochemical pregnancy).

### The Main Outcome Measures

BPL was defined as pregnancy loss after a confirmation of biochemical pregnancy.

CPL was defined as pregnancy loss after a confirmation of clinical pregnancy.

### Endometrial Preparation Protocols

#### Modified Natural Cycle

On the 10th–12th day of the menstrual cycle, follicles were monitored by transvaginal ultrasonography. When the dominant follicle reached a mean diameter of >17 mm, serum LH was <20 IU/L, endometrial thickness reached =>8 mm, and endometrium presented as pattern A, human chorionic gonadotropin (hCG, 10,000 IU, Lizhu Pharmaceutical Trading Co., China) was used as a trigger. Two days after the hCG trigger, patients were administered progesterone.

#### Artificial Cycle

On the first day of the menstrual cycle, patients received estradiol valerate tablets at a dose of 2 mg for 3 days, 4 mg for 6 days, and subsequently 6 mg for 5 days. On the 15th day of the menstrual cycle, transvaginal ultrasound was performed to ascertain the endometrial thickness and pattern. If endometrial thickness reached =>8 mm and endometrium presented as pattern A, patients were administered progesterone. If endometrium presented as pattern A while endometrial thickness reached <8 mm, patients would still take oral estradiol valerate tablets until the endometrial thickness reached =>8 mm.

#### Ovarian Stimulation Cycle

On the 3rd–5th day of the menstrual cycle, patients were administered letrozole (Hengrui Medicine Co., China) for 5 consecutive days. On the 10th day of menstrual cycles, if the diameter of the dominant follicle was <14 mm, human menopausal gonadotropin (Fengyuan Pharmaceutical Co. Anhui, China, 75 IU) was added every other day. When the dominant follicle reached a mean diameter of >17 mm, serum LH was <20 IU/L, endometrial thickness reached =>8 mm, and endometrium presented as pattern A, hCG was used (10,000 IU, Lizhu Pharmaceutical Trading Co., China) as a trigger. Two days after the hCG trigger, patients were administered progesterone.

### Statistical Analysis

All analyses were performed using SPSS software (Version 21, IBM). For a comparison of the constituent ratio, the chi-square test was employed; for a comparison of the continuous data, the data were first examined by the normality and lognormality test. If data fit the pattern of normal distribution, Student’s t test was employed; if not, the Mann–Whitney U test was employed.

In the present study, for **Table 3**, there were 9.6 events per independent variable (EPV) for the analysis of factors affecting BPL, while there were 14 EPVs for the analysis of factors affecting CPL. For **Table 4**, there were 19.7 EPVs for analysis of factors affecting BPL in manner a and 18.1 EPVs for analysis of factors affecting BPL in manner b; there were 24.9 EPVs for analysis of factors affecting CPL in manner a and 22.8 EPVs for analysis of CPL in manner b. According to the principle of 10 EPVs (17), the sample size in the present study was appropriate.

**Table 3:** Association between factors and BPL/CPL for frozen-thawed blastocyst transfer.

	Biochemical pregnancy loss		Clinical pregnancy loss	
	OR (95% CL)	p	OR (95% CL)	p
Age				
Female	1.046 (0.964–1.135)	0.282	1.019 (0.950–1.094)	0.596
Male	1.005 (0.950–1.063)	0.870	1.036 (0.988–1.086)	0.148
BMI				
Female	0.994 (0.941–1.049)	0.814	1.028 (0.982–1.075)	0.238
Male	1.015 (0.962–1.071)	0.584	1.004 (0.959–1.052)	0.864
Semen DFI	0.996 (0.969–1.022)	0.743	0.997 (0.974–1.020)	0.775
History of miscarriage				
No	1 (ref)		1 (ref)	
Yes	0.280 (0.037–2.104)	0.216	3.767 (1.769–8.021)	0.001
Number of embryos transferred	0.681 (0.440–1.054)	0.085	0.760 (0.524–1.103)	0.149
Endometrial preparation				
Ovarian stimulation cycle	1 (ref)		1 (ref)	
Modified natural cycle	0.548 (0.152–1.979)	0.359	1.726 (0.569–5.237)	0.335
Artificial cycle	0.590 (0.367–0.948)	0.029	1.555 (0.921–2.625)	0.099
Type of embryos transferred				
Group A (n=984)	1 (ref)		1 (ref)	
Group B (n=178)	1.774 (1.053–2.990)	0.031	1.735 (1.065–2.828)	0.027
FET cycles with days of embryos				
Day 5 (n=740)	1 (ref)		1 (ref)	
Day 6 (n=355)	1.930 (1.247–2.987)	0.003	1.639 (1.127–2.384)	0.001

Data are presented as the OR (95% CL).

DFI, DNA fragmentation index; OR, odds ratio; FET, frozen-thawed embryo transfer.

**Table 4:** Association between factors and BPL/CPL for frozen-thawed blastocyst and day 3 cleavage embryo transfer.

	Biochemical pregnancy loss		Clinical pregnancy loss	
	OR (95% CL)	p	OR (95% CL)	p
^a^Groups				
Group C (n=672, 78%)	1 (ref)		1 (ref)	
Group A (n=984, 100%)	0.497 (0.350–0.705)	0.000	0.743 (0.538–1.025)	0.071
Group B (n=178, 39.7%)	1.134 (0.723–1.779)	0.585	1.425 (0.913–2.226)	0.119
^a^Age				
Female	1.041(0.980–1.106)	0.194	1.020 (0.964–1.079)	0.485
Male	1.006 (0.965–1.050)	0.766	1.036 (0.996–1.077)	0.076
^a^BMI				
Female	0.996 (0.958–1.035)	0.829	1.028 (0.994–1.064)	0.109
Male	1.024 (0.983–1.066)	0.264	1.013 (0.975–1.052)	0.521
^a^Semen DFI	1.004 (0.986–1.024)	0.650	1.001 (0.983–1.018)	0.943
^a^History of miscarriage				
No	1 (ref)		1 (ref)	
Yes	0.989 (0.437–2.240)	0.979	4.014 (2.221–7.253)	0.000
^a^Number of embryos transferred	0.795 (0.569–1.111)	0.179	0.824 (0.607–1.118)	0.214
^a^Endometrial preparation				
Ovarian stimulation cycle	1 (ref)		1 (ref)	
Modified natural cycle	0.752 (0.333–1.700)	0.494	2.112 (1.023–4.362)	0.043
Artificial cycle	0.684 (0.482–0.791)	0.033	1.248 (0.856–1.819)	0.250
^b^FET cycles with days of embryos				
Group C (n=672, 53.9%)	1 (ref)		1 (ref)	
Day 5 (n=740, 100%)	0.420 (0.285–0.620)	0.000	0.697 (0.494–0.983)	0.039
Day 6 (n=355, 0%)	0.914 (0.623–1.340)	0.645	1.248 (0.866–1.799)	0.234

Data are presented as the OR (95%CL).

DFI, DNA fragmentation index; OR, odds ratio; FET, frozen-thawed embryo transfer.

For groups, group name (n=cases, the percentage of MG blastocysts or predicted MG blastocysts).

For FET cycles with days of embryos (n=cases, the percentage of day 5 blastocysts or predicted day 5 blastocysts).

a Age or BMI of females or males, semen DFI, number of embryos transferred, endometrial preparation, history of miscarriage, and groups were included for analysis.

b Age or BMI of females or males, semen DFI, number of embryos transferred, endometrial preparation, history of miscarriage, and days of embryos were included for analysis. Only results of days of embryos were presented. The results of other indicators in b were similar with those in a.

Binary logistic regression analyses were used to evaluate the association between factors (including MA of blastocysts, days of blastocysts, developmental stage of embryos, age of males and females, BMI of females and males, semen DFI, and protocols of endometrial preparation) and BPL or CPL.

A p value of <0.05 was considered a statistically significant difference.

## Results

### Baseline Characteristics of Patients and FET Cycles

According to the *in vitro* cultural data of Day 3 cleavage embryos, the ratio of usable MG blastocysts formed from grade I embryos was 88.9%, from grade II embryos was 78.5%, and from grade III embryos was 45.7%, while the ratio of usable Day 5 blastocysts formed from grade I embryos was 62.7%, from grade II embryos was 54.2%, and from grade III embryos was 28.9% **(** **Table 5** **)**. Here, we assumed that all the Day 3 embryos transferred in the present study formed useable blastocysts *in vivo*. This was a rough evaluation without taking the “unusable blastocysts” for consideration. Based on these results, we predicted that 78% of *in vivo*-formed MG blastocysts and 53.9% of *in vivo*-formed Day 5 blastocysts resulted from the transfer of Day 3 cleavage embryos in the present study (**Table 6**).

**Table 5:** The ratio of formed MG or day 5 blastocysts to formed usable blastocysts from extended culture of different grading of Day 3 embryos.

	I	II	III
Usable blastocysts^#^	1,197	10,621	1372
MG blastocyst (%)	1,064 (88.9)	8,336 (78.5)	627 (45.7)
Day 5 blastocyst (%)	751 (62.7)	5,752 (54.2)	397 (28.9)

Data are presented as the count (percentage).

MG, morphologically good; MNG, morphologically non-good.

^#^Usable blastocysts were equal to MG blastocysts plus MNG blastocysts, or equal to day 5 MG and MNG blastocysts plus day 6 MG and MNG blastocysts.

**Table 6:** Predicted *in vivo*-formed number and ratio of MG and Day 5 blastocysts to *in vivo*-formed usable blastocysts for Day 3 cleavage embryos transferred in the present study.

	I	II	III	Total
Day 3 embryos Transferred	183	1,004	77	1,264
Predicted number of MG blastocysts	162.6	788.1	35.2	985.9
Predicted number of day 5 blastocysts	114.7	544	22.2	680.9
Predicted rate of MG blastocysts (%)	–	–	–	985.0/1,264 (78.0)
Predicted rate of day 5 blastocysts (%)	–	–	–	680.9/1,264 (53.9)

Data are presented as the count (percentage).

We assumed that all the embryos transferred can form usable blastocysts, and the in vitro and in vivo developmental potential of Day 3 cleavage embryos were similar.

The percentage of primary infertility, primary diagnosis of tubal factors, endometriosis, and male factors, FET cycle-originated fresh cycles with GnRH antagonist, previous miscarriage, single implantation and singleton, BMI of males and females, and infertility duration were comparable among groups (**Table 7**). The percentage of MG blastocysts and Day 5 blastocysts transferred, the percentage of primary diagnosis of DOR, the percentage of FET cycle-originated fresh cycles with GnRH agonist/without GnRH analogs, the age of males and females at oocyte retrieval, the female age at embryo transfer, the semen DFI, the number of embryos transferred, the protocols of endometrial preparation, and the rate of BPL and CPL among groups were significantly different (**Table 7**). Furthermore, the percentage of FET cycles with different days of blastocysts between Group A and Group B was significantly different (**Table 7**).

**Table 7:** Characteristics of patients and FET cycles.

	Group A	Group B	Group C	p1	p2	p3	p4
FET cycles	984	178	672				
MG blastocysts (%)	1,417/1,417 (100)	131/330 (39.7)	985.9/1,264 (78.0)^*^	0.000	0.000	0.000	0.000
Day 5 blastocysts (%)	1,015/1,417 (71.6)	110/330 (33.3)	680.9/1,264 (53.9)^#^	0.000	0.000	0.000	0.000
Primary infertility (%)	575 (58.43)	110 (61.80)	408 (60.71)	0.533	0.401	0.354	0.792
Infertility duration	3.24 ± 1.95	3.23 ± 2.12	3.40 ± 2.29	0.280	0.965	0.125	0.340
Primary diagnosis (%)							
Tubal factor	481 (48.89)	89 (50.0)	302 (44.94)	0.227	0.784	0.115	0.228
Endometriosis	36 (3.66)	7 (3.93)	38 (5.65)	0.144	0.859	0.054	0.362
DOR	19 (1.93)	1 (0.56)	76 (11.31)	0.000	0.196	0.000	0.000
Male factor	143 (14.53)	31 (17.42)	88 (13.10)	0.325	0.321	0.407	0.140
Others	305 (30.99)	50 (28.1)	168 (25)	0.029	0.439	0.008	0.401
GnRH analogues							
Agonist	764 (77.64)	143 (80.33)	327 (48.66)	0.000	0.424	0.000	0.000
Antagonist	169 (17.17)	28 (15.73)	140 (20.83)	0.106	0.636	0.061	0.128
No analogues	51 (5.18)	7 (3.93)	205 (30.51)	0.000	0.481	0.000	0.000
Age at oocyte retrieval							
Female	29.44 ± 3.11	29.42 ± 3.26	30.06 ± 3.17	0.000	0.873	0.000	0.014
Male	31.45 ± 4.42	31.56 ± 4.59	31.99 ± 4.32	0.048	0.903	0.027	0.233
Female age at embryo transfer	29.83 ± 3.15	29.88 ± 3.33	30.51 ± 3.21	0.000	0.907	0.000	0.017
BMI							
Female	22.57 ± 3.57	22.82 ± 3.96	22.8 ± 3.85	0.397	0.410	0.211	0.957
Male	24.92 ± 3.70	25.05 ± 3.70	24.76 ± 3.19	0.529	0.643	0.379	0.332
Semen DFI	12.47 ± 7.23	14.03 ± 7.97	13.73 ± 7.64	0.001	0.010	0.001	0.635
FET cycle with days of blastocysts							
Day 5	697/984 (70.8)	43/178 (24.2)	–	–	0.000	–	–
Day 5+6	38/984 (3.9)	29/178 (16.3)	–	–	0.000	–	–
Day 6	249/984 (25.3)	106/178 (59.6)	–	–	0.000	–	–
Previous miscarriage	31/984 (3.2)	1/178 (0.6)	25/672 (3.7)	0.097	0.090	0.529	0.030
Number of embryos transferred	1.44 ± 0.50	1.85 ± 0.37	1.88 ± 0.36	0.000	0.000	0.000	0.420
Single implantation (%)	685/899 (76.2)	105/148 (70.9)	411/570 (72.1)	0.135	0.169	0.079	0.780
Singleton (%)	605/763 (79.3)	86/115(74.8)	360/465 (77.4)	0.475	0.271	0.438	0.548
Endometrial preparation (%)							
Modified natural cycle	25/984 (2.54)	5/178 (2.81)	31/672 (4.61)	0.064	0.837	0.022	0.288
Artificial cycle	813/984 (82.62)	134/178 (75.28)	514/672 (76.49)	0.003	0.020	0.002	0.737
Ovarian stimulation cycle	146/984 (14.84)	39/178 (21.91)	127/672 (18.90)	0.018	0.018	0.029	0.368
Biochemical pregnancy loss (%)	85/984 (8.6)	30/178 (16.9)	102/672 (15.2)	0.000	0.001	0.000	0.583
Clinical pregnancy loss (%)	136/899 (15.1)	33/148 (22.3)	105/570 (18.8)	0.05	0.028	0.097	1.137

Data are presented as the mean+SD or count (percentage).

FET, frozen-thawed embryo transfer; MG, morphologically good; MNG, morphologically non-good; DOR, declined ovarian reserve; DFI, DNA fragmentation index.

*Predicted rate of MG blastocysts in vivo from transfer of day 3 embryos.

^#^Predicted rate of day 5 blastocysts in vivo from transfer of day 3 embryos.

p1, comparison among the three groups; p2, group A vs. group B; p3, group A vs. Group C; p4, group B vs. group C.

### MA or Days of Blastocysts Were Associated With BPL or CPL

The age or BMI of females or males, semen DFI, and number of embryos transferred had no influence on the occurrence of BPL and CPL (**Table 3**). A history of miscarriage significantly and positively affected the occurrence of CPL, not BPL (**Table 3**). Compared to the ovarian stimulation cycle, the artificial cycle showed a significantly reduced rate of BPL (**Table 3**). Group B had significantly higher rates of BPL and CPL than Group A (**Table 3**). Similarly, FET cycles with Day 6 blastocysts had significantly higher rates of BPL and CPL than FET cycles with Day 5 blastocysts (**Table 3**).

### Comparison of BPL or CPL Between Transfer of Blastocysts and Transfer of Day 3 Cleavage Embryos

After adding Group C for analysis, we also found that age or BMI of females or males, semen DFI, and number of embryos transferred had no influence on the occurrence of BPL or CPL, and a history of miscarriage significantly and positively affected the occurrence of CPL, not BPL (**Table 4**). Compared to the ovarian stimulation cycle, the artificial cycle showed a significantly reduced rate of BPL, while the natural cycle showed a significantly higher rate of CPL (**Table 4**). After controlling factors including age and BMI of females and males, semen DFI, number of embryos transferred, and protocols of endometrial preparation, we found that Group C had a significantly higher rate of BPL than Group A (p=0.00) and a similar rate of BPL as Group B (p=0.585) (**Table 4**). However, Group C had a higher rate of CPL than Group A (p=0.071) and a reduced rate of CPL compared to Group B (p=0.119) (**Table 4**). Similarly, after controlling factors including age or BMI of females or males, semen DFI, and number of embryos transferred and protocols of endometrial preparation, we found that Group C had a significantly higher rate of BPL than FET cycles with Day 5 blastocysts (p=0.000) and a similar rate of BPL as FET cycles with Day 6 blastocysts (p=0.645) (**Table 4**). However, Group C had a significantly higher rate of CPL than FET cycles with Day 5 blastocysts (p=0.039) and a reduced rate of CPL compared to FET cycles with Day 6 blastocysts (p=0.24) (**Table 4**).

**Figure 2:**
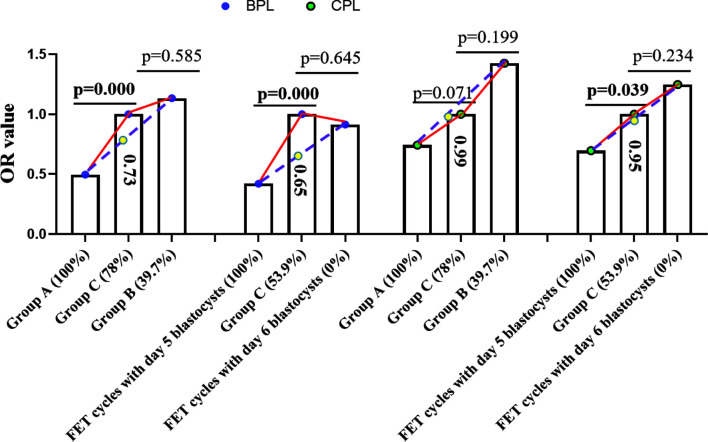
Prediction of the OR value of Group C. According to the OR value of Group A and Group B or the OR value of FET cycles with Day 5/Day 6 blastocysts and the rate of MG blastocysts in Group A and Group B or the rate of Day 5 blastocysts in FET cycles with Day 5 and Day 6 blastocysts, we predicted the OR value of Group C (the predicted OR value is in the column of Group C). The blue circle indicates the OR value of BPL, the green circle indicates the OR value of CPL, and the yellow circle indicates the predicted OR value of BPL or CPL in group C. Values in brackets indicate the percentage of MG or Day 5 blastocysts in each group. BPL, biochemical pregnancy loss; CPL, clinical pregnancy loss; MG, morphologically good; MNG, morphologically non-good; FET, frozen embryo transfer; OR, odds ratio.

We further tested whether the OR value of Group C (compared to Groups A and B or FET cycles with Day 5 blastocysts and Day 6 blastocysts) fitted the findings from the transfer of blastocysts, and we performed some analyses. The rates of MG blastocysts in Groups A, C, and B were 100 *vs.* 78 *vs.* 39.7%, while the rates of Day 5 blastocysts in Groups A, C, and B were 100 *vs.* 53.9 *vs.* 0% **(** **Figure 2** **)**. Considering that MA and days of blastocysts correlated with the occurrence of BPL and CPL for the transfer of blastocysts, we assumed a linear relationship between the percentage of MG or Day 5 blastocysts and the rate of BPL or CPL in each group. According to the rate of MG or Day 5 blastocysts and the corresponding rate of BPL or CPL for transfer of blastocysts, we predicted the OR value in Group C. For BPL, the predicted OR value (yellow circle) for Group C was obviously lower than the observed OR value grouped by either MA (0.73 *vs.* 1) or days of blastocyst (0.65 *vs.* 1); for CPL, the predicted OR value (yellow circle) for Group C was similar to the observed OR value grouped by either MA (0.99 *vs.* 1) or days of blastocyst (0.95 *vs.* 1) **(** **Figure 2** **).** Obviously, CPL, not BPL, resulting from the transfer of Day 3 embryos met our expectation.

## Discussion

The argument of pregnancy loss between the transfer of cleavage embryos and blastocysts needs to be clarified. In the present study, our results indicated that the quality of blastocysts obviously affected the occurrence of BPL or CPL, and the difference in the rate of BPL or CPL between transfer of blastocysts and Day 3 cleavage embryos depends on the quality of blastocysts and cleavage embryos transferred. In addition, our study also highlights the increased rate of BPL in the transfer of Day 3 cleavage embryos.

There is consensus that blastocysts are a specific embryo stage that initiates the interaction between embryos and utero (1). Viewed from the perspective of embryos, at least four events account for a failed live birth resulting from transfer of a Day 3 cleavage embryo in IVF: (a) Embryos cannot develop into the blastocyst stage. (b) Blastocyst fails to interact with utero. (c) Blastocyst fails to complete implantation. (d) Abortion. The DP of embryos increased from a to d; c and d were investigated in the present study. Therefore, only β-HCG-positive FET cycles were included in the present study.

In IVF, Day 3 cleavage embryos and blastocysts are the most commonly used for transfer. The biggest difference between the transfer of Day 3 embryos and blastocysts is the further development of Day 3 embryos. For transfer of Day 3 embryos, this development occurs *in utero*, while for transfer of blastocysts, development occurs in the culture medium. Extended culture allows us to evaluate blastocysts and select blastocysts of good quality for transfer. However, it is impossible to evaluate the blastocysts *in vivo* when performing transfer of Day 3 embryos. In the present study, we summarized the data of extended culture in our reproductive center and predicted 78% of *in vivo*-formed MG blastocysts and 53.9% of Day 5 blastocysts for transfer of Day 3 cleavage embryos (Group C). This prediction was based on the assumptions that the *in vitro* and *in vivo* blastulation abilities of Day 3 embryos were similar, and all transferred Day 3 embryos formed usable blastocysts and resulted in biochemical pregnancy.

Because few embryos could be retrieved from patients with declined ovarian reserve (DOR) and extended culture may result in no embryos for transfer for those patients, patients with DOR were prone to receive Day 3 cleavage embryo transfer. This is why there were more patients with DOR in Group C. Consequently, these patients were treated with mild ovarian stimulation protocols. A previous study demonstrated that oocytes from young women with normal and decreased ovarian reserve show similar quality (18). Therefore, we deduced that decreased ovarian reserve may not have influenced BPL or CPL in the present study.

Many factors that were reported to possibly affect the occurrence of BPL or CPL were taken as confounding factors for analysis in the present study, including age at oocyte retrieval/BMI of males or females (19–25), semen DFI (26, 27), history of miscarriage (21, 28), number of embryos transferred (29–31), and endometrial preparation protocols (32). In the present study, we found that most of these factors were not related to the occurrence of BPL or CPL. However, we ascertained that a history of miscarriage was tightly associated with the occurrence of CPL but not BPL, indicating the difference between the occurrence of BPL and CPL. A previous study showed that the pregnancy loss rate was significantly higher with ovarian stimulation cycles or artificial cycles than with natural cycles (32). However, our study supported the use of artificial endometrial preparation. The most commonly used endometrial preparation protocol in our center was artificial cycles, and cases in the natural cycle or ovarian stimulation cycles were very limited. Therefore, this conclusion still needs to be verified by large datasets.

To our knowledge, no studies have been conducted to investigate whether MA or days of blastocysts can predict BPL. In the present study, we found that Group B and FET cycles with Day 6 blastocysts had significantly higher rates of BPL than Group A and FET cycles with Day 5 blastocysts, respectively. Due to limited cases of β-HCG-positive FET cycles with MNG blastocysts only, we included FET cycles with at least one MNG blastocyst. Group B had 39.7% of MG blastocysts. Therefore, we deduced that FET cycles with MNG blastocysts only may further increase the rate of BPL. These results indicated that MA or days of blastocyst could serve as an independent factor affecting the occurrence of BPL.

Inconsistent with our prediction, we found that Group C had a significantly higher rate of BPL than Group A or FET cycles with Day 5 blastocysts and a similar rate of BPL as Group B or FET cycles with Day 6 blastocysts. However, this result was consistent with a previous study showing that early pregnancy loss was significantly higher after Day 3 single embryo transfer than after Day 5 single blastocyst transfer (33). For blastocyst transfer, usable blastocysts (only MG or MNG blastocysts) will be selected for use. In contrast, for transfer of Day 3 cleavage embryos, the cleavage embryos not only produce usable blastocysts but also “unusable blastocysts” *in vivo*. Several studies have demonstrated the DP of “unusable blastocysts”, including MB and Day 7 blastocysts (34–36). Therefore, for Day 3 embryo transfer, biochemical pregnancy may result not only from MG or MNG blastocysts but also from MB or Day 7 blastocysts in the present study. Previous studies revealed that the rate of BPL was similar between transfer of MB or Day 5 blastocysts and transfer of MB or Day 7 blastocysts (34, 37). In their studies, the rate of BPL was defined as the number of FET cycles with BPL to total FET cycles, not to β-HCG-positive FET cycles. Obviously, this definition is not scientific and correct, as most Day 7 or MB blastocysts will not result in biochemical pregnancy. Therefore, the rate of BPL should be defined as the rate of FET cycles with BPL to β-HCG-positive FET cycles. After reanalyzing the data in the two published articles, we found that the rate of BPL of transfer of MB/Day 7 blastocysts was significantly higher (3 times) than that of the transfer of MG/Day 5 blastocysts. Therefore, we deduced that the biochemical pregnancy resulting from *in vivo*-formed unusable blastocysts may account for the higher BPL for the transfer of Day 3 cleavage embryos. This could explain the similar rate of BPL between transfer of MNG or Day 6 blastocysts and transfer of Day 3 cleavage embryos in the present study.

Numerous studies have been conducted to investigate the role of MA or days of blastocysts in predicting clinical pregnancy and live birth (38–40). Few studies have focused on investigating the effect of MA or the day of blastocyst on the occurrence of pregnancy loss. Previous studies have investigated the relationship between the quality of TE or ICM and miscarriage; however, no consistent conclusions have been drawn (41, 42). A recent study reported no statistical correlation between miscarriage and the grade of ICM or TE (43). In that study, they only included blastocysts with grade A or B ICM and without grade C ICM; therefore, it is not strange that no association of ICM grade with miscarriage was observed (43). However, that study showed a trend that the C score of TE was related to a higher rate of miscarriage (43). A common drawback of these studies is that they only considered a part of the blastocyst (TE or ICM) and did not take the blastocyst as a whole for analysis. However, our morphological classification of blastocysts in the present study could avoid this. In the present study, we found that Group B and FET cycles with Day 6 blastocysts had a significantly higher rate of CPL than Group A and FET cycles with Day 5 blastocysts, respectively. Part of these results was consistent with previous reports that transfer of Day 6 blastocysts resulted in a significantly higher miscarriage rate than that of Day 5 blastocysts (43, 44). These results indicated that MA or days of blastocyst could serve as an independent factor affecting the occurrence of CPL.

Consistent with our prediction, we found that Group C had a higher rate of CPL than Group A and FET cycles with Day 5 blastocysts and a lower rate of CPL than Group B and FET cycles with Day 6 blastocysts. However, some comparisons failed to reach statistical significance. Similar to biochemical pregnancy, it is possible that MB or Day 7 embryos contributed to clinical pregnancy for the transfer of Day 3 embryos in the present study. However, we did not observe an obviously higher rate of CPL for transfer of Day 3 embryos, indicating that the possibly implanted “unusable blastocysts” produced by Day 3 cleavage embryos *in vivo* did not result in a significantly higher rate of CPL compared to transfer of MG or Day 5 blastocysts. This is consistent with previous studies reporting that the CPL between transfer of MG/Day 5 blastocysts and transfer of MB/Day 7 blastocysts was similar (34, 37). However, due to the too limited implantation ability of “unusable blastocysts”, whether “unusable blastocysts” would not result in a significantly higher rate of CPL remains to be investigated by using large data.

Taking all the results as a whole for analysis, we concluded that (1) MA or days of blastocyst served as an independent factor affecting the occurrence of BPL and CPL. (2) Transfer of Day 3 cleavage embryos may result in “unusable blastocysts”, which may significantly increase the rate of BPL. (3) The rate of CPL resulting from the transfer of Day 3 embryos may depend on the rate of *in vivo*-formed MG or Day 5 blastocysts (to *in vivo*-formed useable blastocysts). Therefore, when comparing the rate of BPL or CPL between transfer of blastocysts and Day 3 cleavage embryos, the quality of embryos transferred, including blastocysts or cleavage embryos, should be taken into consideration. Our study suggested that morphologically good Day 5 blastocysts should be first considered for transfer, not only because of the high implantation potential, as a previous study reported (45), but also because of the low rate of pregnancy loss. Furthermore, our study may help to explain a recently published study indicating that FET cycles with blastocysts arising from poor-quality cleavage embryos showed a significantly higher miscarriage rate (46). As we showed in the present study, the rate of Day 5 blastocysts from grade III Day 3 embryos was 29%, while that from grade I embryos was 62.7%. Although the MA of transferred blastocysts among groups was similar in that study, it was more likely that Day 5 blastocysts were selected for transfer in the grade I group, thereby resulting in a difference in the miscarriage rate. The comprehensive analysis of the present study advanced our understanding of the occurrence of BPL and CPL between transfer of blastocysts or Day 3 cleavage embryos. Therefore, our study is clinically and theoretically important.

Due to the retrospective nature of the present study, limitations were presented, including possible confounding factors that were not considered in the present study. Our study included FET cycles with double embryos, which may introduce bias. FET cycles with two embryos may result in two implantations and one live birth. However, these FET cycles were not taken as cycles with CPL or possible BPL. Furthermore, the sample size was not large enough to reach statistical significance for some comparisons. Additionally, data from a single center may dampen the confidence of the present study. Therefore, our study needs to be confirmed by a large study.

## Conclusion

We concluded that (1) MA or days of blastocyst served as an independent factor affecting the occurrence of BPL or CPL. (2) Transfer of Day 3 cleavage embryos may produce “unusable blastocysts”, which may significantly increase the rate of BPL. (3) The rate of CPL resulting from the transfer of Day 3 embryos may depend on the rate of *in vivo*-formed MG or Day 5 blastocysts. In conclusion, the difference in BPL or CPL between transfer of blastocysts and Day 3 cleavage embryos depends on the quality of embryos transferred. Our study advanced our understanding of the occurrence of BPL and CPL between transfer of blastocysts and Day 3 cleavage embryos.

## Data Availability Statement

The original contributions presented in the study are included in the article. Further inquiries can be directed to the corresponding author.

## Ethics Statement

All of the included patients read and signed the informed consent form. This retrospective study was approved by the Ethics Committee of Changzhou Maternal and Child Health Care Hospital and Nanjing Medical University.

## Author Contributions

Substantial contribution to conception and design: LC, XD, and YW. Data acquisition: TG, XX, and FC. Data analysis: TG, XX, FC, CY, TL, and LL. Data interpretation: All authors. Drafting the article: XD and YW. Critical revision of the article for important intellectual content: All authors. All authors contributed to the article and approved the submitted version.

## Funding

This study was supported by the National Natural Science Foundation of China (No. 81901436, to XD), Changzhou Health Committee Funded Young Investigator Training Project (CZQM2020094 to XD and CZQM2020099 to TG), Key Program of Changzhou Municipal Health Commission (ZD201921 to LC), Program of Jiangsu Province’s Key Provincial Talents of Women and Child Health Care (FRC 201751 to LC), and Jiangsu Population Institute funded program (JSPA2019017 to TL).

## Conflict of Interest

The authors declare that the research was conducted in the absence of any commercial or financial relationships that could be construed as a potential conflict of interest.

## Publisher’s Note

All claims expressed in this article are solely those of the authors and do not necessarily represent those of their affiliated organizations, or those of the publisher, the editors and the reviewers. Any product that may be evaluated in this article, or claim that may be made by its manufacturer, is not guaranteed or endorsed by the publisher.
